# Correction: Sexual dysfunction among men with diabetes mellitus attending chronic out-patient department at the three hospitals of Northwest Amhara region, Ethiopia: Prevalence and associated factors

**DOI:** 10.1371/journal.pone.0271856

**Published:** 2022-07-18

**Authors:** Eskedar Getie Mekonnen, Hedija Yenus Yeshita, Alehegn Bishaw Geremew

There is an error in affiliation 1 for authors Eskedar Getie Mekonnen, Hedija Yenus Yeshita, and Alehegn Bishaw Geremew. The correct affiliation 1 is: Department of Reproductive Health, Institute of Public Health, College of Medicine and Health Sciences, University of Gondar, Gondar, Ethiopia.
