# Supplementary material for: Sexual dysfunction among men with diabetes mellitus attending chronic out-patient department at the three hospitals of Northwest Amhara region, Ethiopia: Prevalence and associated factors
Source: PLoS One. 2021 Aug 11;16(8):e0256015. doi: 10.1371/journal.pone.0256015 (PMC8357135; doi:10.1371/journal.pone.0256015)
Supplement: Attachment — Submitted filename: Response to Reviewers.docx [file pone.0256015.s002.docx]

**May 18/2021**

**Point-by-point response**

Dear the editor and reviewers, we found your comments to be crucial for enhancing our scholarly work. We are really grateful enough to express our appreciation for your comments. Appreciating your effort and valuable comments, we have provided possible reflections on the raised concerns and questions. Kindly find our response hereunder.

A. **Editor’s comment**
1. Please ensure that your manuscript meets PLOS ONE's style requirements, including those for file naming.

**Authors’ response:** comment has been accepted.

2. In the Methods, please clarify that participants provided oral consent. Please also state in the Methods: why written consent could not be obtained? Whether the Institutional Review Board (IRB) approved use of oral consent?, and how oral consent was documented?

**Authors’ response:** Dear, the reason why we could not obtain written consent was as the study never used biological samples from the participants and applied any invasive procedure, we have taken oral informed consent that approved by the Institutional review board committee of the University of Gondar.

As per your recommendation, the ethical approval statement has appeared only in the method section of the manuscript and the detail of ethical consideration was presented in the updated version.

3. Please include additional information regarding the survey or questionnaire used in the study and ensure that you have provided sufficient details that others could replicate the analyses. For instance, if you developed a questionnaire as part of this study and it is not under a copyright more restrictive than CC-BY, please include a copy, in both the original language and English, as Supporting Information.

**Author’s response:** Dear, we had collected the data for the outcome variable using the Changes in Sexual Functioning Questionnaire (CSFQ-14) adapted from the changes in sexual functioning questionnaire short form (CSFQ14) that any scholar can access it online. Similarly, for the independent variables, daily stressful event measurement scale (DSEMS) and relationship satisfaction (CSI), for instance, were taken from the previously published articles that were cited in the main document. We can supply a copy of both the Amharic (local language) and English language tool that we used to collect the data if that is necessary.

4. Please ensure you have thoroughly discussed any additional potential limitations of this study within the discussion section, including the potential impact of confounding factors.

**Author’s response:** comment accepted and the potential limitation of the study has been added in the newly revised manuscript.

5. We suggest you thoroughly copyedit your manuscript for language usage, spelling, and grammar. If you do not know anyone who can help you do this, you may wish to consider employing a professional scientific editing service.

**Author’s response:** comment accepted and the language usage has been improved.

6. Your ethics statement should only appear in the Methods section of your manuscript. If your ethics statement is written in any section besides the Methods, please delete it from any other section.

**Author’s response:** comment accepted and the necessary modification has been undertaken.

B. **Reviewer #1**
   1. The manuscript is seriously flawed by the poor quality of the English used.
      I suggest an extensive revision of the English by a specialist to correct the enormous number of mistakes in grammar and typos.

**Authors’ response**: comment accepted and necessary modification has been done.

2. The last three sentences of the introduction should be move to the discussion

**Author’s comment:** comment accepted and the statements have been taken to the discussion section.

3. Details on the CSFQ should be transferred in supplementary files to shorten the methods section.

**Author’s comment:** we appreciate your concern dear, and we have precisely presented it in the methods section.

4. In the methods, definition of the microvascular complications of diabetes should be given (retinopathy, nephropathy, neuropathy)

**Authors’ comment:** comment accepted and it has been modified accordingly.

5. Do the authors measure the testosterone levels of the participants in the study? Have hypogonadic men been excluded?

**Authors’ comment:** Dear, the higher burden of hypogonadism among type II diabetic patients was previously evidenced, which could be one of a pathological pathway for the development of SD. We believe that excluding hypogonadic men in our study would largely affect our study because of introducing bias, in particular, selection bias. Patients with SD might obviously have hypogonadism and excluding these people would severely affect the outcome variable and lead to miss inference. Although measuring the level of testosterone is truly important, we didn’t measure it as the test was not available in the study setting and even in our country.

6. Have the interference of drugs interfering with sexual function (i.e. beta-blockers, anti-depressants, etc.) been considered?

**Authors’ comment:** the existence of other comorbidities along with their medication, as well as other drugs with a possible side effect of SD was examined. The data were collected through reviewing the medical recording of participants as those factors might have confounding effect. Then the medication history was considered to include in the analysis but it doesn’t fulfil the chi-square assumption. We have included the descriptive data on the currently updated document.

C. **Reviewer #2**
   1. This cross-sectional study was conducted among 462 diabetic men in Ethiopia, and sexual dysfunction was found in 69.5% based on questionnaires collected. No control group was included, which should be underlined as a major limitation. However, due to the high number of patients included, the manuscript deserves publication after revision.

**Authors’ comment:** Dear, as our primary objective was to investigate the prevalence of SD among men with diabetes, we didn’t consider a control group. Dear, if we had established a control (patient without SD) and case group (participants with SD), the result would have differed from our primary objective (prevalence of SD) and we wouldn’t have estimated the prevalence. Thus, we don’t believe that not considering the control group wouldn’t be the possible limitation of the study. However, we are ready to accept your comment if you are not satisfied with the feedback given.

2. Abstract - Results, line 2: Should "disproportionately" be replaced by "proportionately"?

**Author’s comment**: comment accepted and corrected accordingly.

3. Abstract - Conclusion, line 2: Should "physical activity" be replaced by "lack in physical activity"?

**Authors’ comment:** comment accepted and modified accordingly

4. Main text - Background, line 28: "hypertension increased the problem". Are you sure it is the hypertension itself? Could erectile dysfunction rather be caused by the anti-hypertension treatment according to e.g. Fedder et al., 2013?

**Authors comment:** SD among hypertensive patients are multifactorial. Not only antihypertensive medications have a deleterious effect on an individual’s sexual function rather hypertension by itself have an impact on patients sexual function associated with its effect on the blood vessels of the genitalia. Likewise, as you said, it’s undeniable evidence that anti-hypertensive agents like β-blockers and diuretics could have the potential to further impair an individual’s sexual function through reducing blood flow to the reproductive organs. Moreover, the psychological impact of chronic illnesses are another contributing factor for developing SD. All in all, you are right both factors (hypertension and the drugs) are responsible and they have been included in the study and examined for its association with SD.

5. Reference list: For at least 10 references, the name of the journal is not mentioned. It should be included.

**Authors’ comment:** comment accepted and the names of the journals have been included except for the gray literatures.
